# Mimicking Pseudo-Virion Interactions with Abiotic Surfaces: Deposition of Polymer Nanoparticles with Albumin Corona

**DOI:** 10.3390/biom12111658

**Published:** 2022-11-08

**Authors:** Małgorzata Nattich-Rak, Marta Sadowska, Maja Motyczyńska, Zbigniew Adamczyk

**Affiliations:** 1 Jerzy Haber Institute of Catalysis and Surface Chemistry, Polish Academy of Sciences, Niezapominajek 8, 30-239 Cracow, Poland; 2 The Faculty of Biochemistry, Biophysics and Biotechnology, Jagiellonian University, ul. Gronostajowa 7, 30-387 Cracow, Poland

**Keywords:** adsorption of albumin, albumin coronas of particles, deposition of polymer albumin conjugates, stability of albumin corona, virus deposition, zeta potential of albumin corona

## Abstract

Adsorption of human serum albumin (HSA) molecules on negatively charged polystyrene microparticles was studied using the dynamic light scattering, the electrophoretic and the solution depletion methods involving atomic force microscopy. Initially, the physicochemical characteristics of the albumin comprising the hydrodynamic diameter, the zeta potential and the isoelectric point were determined as a function of pH. Analogous characteristics of the polymer particles were acquired, including their size and zeta potential. The formation of albumin corona on the particles was investigated in situ by electrophoretic mobility measurements. The size, stability and electrokinetic properties of the particles with the corona were also determined. The particle diameter was equal to 125 nm, which coincides with the size of the SARS-CoV-2 virion. The isoelectric point of the particles appeared at a pH of 5. The deposition kinetics of the particles was determined by atomic force microscopy (AFM) under diffusion and by quartz microbalance (QCM) under flow conditions. It was shown that the deposition rate at a gold sensor abruptly vanished with pH following the decrease in the zeta potential of the particles. It is postulated that the acquired results can be used as useful reference systems mimicking virus adsorption on abiotic surfaces.

## 1. Introduction

Protein adsorption on particles of various size is essential for their efficient separation and purification by chromatography and filtration, for biosensing, enzymatic catalysis, bioreactors and immunological assays. For example, polymer particles conjugated with various immunoglobulins, referred to as immunolatex [1,2,3,4], are used in a plethora of sensitive immunoassays designed to detect various bacterial infections, for example *Salmonella*, *E. coli*, etc.

On the other hand, blood serum protein adsorption may exert adverse effects on polymer and noble metal (silver and gold) nanoparticles or drug-loaded capsules and liposomes used to deliver therapeutic and contrast imaging agents for cancer diagnosis and treatment, comprising the hyperthermia therapy [5,6,7,8,9,10,11,12,13,14].

Because of its essential significance, adsorption of proteins by nanoparticles, referred to as corona formation, was extensively studied both for single-molecule systems and for mixtures stemming from the blood serum [15,16,17,18,19,20,21,22,23,24,25,26,27]. For larger particles, investigations of albumin and fibrinogen adsorption using the electrokinetic techniques were carried out in Refs. [28,29,30].

It is generally postulated [15,16,17,18,19] that there exists a fraction of irreversibly bound protein forming the hard corona and a fraction of less tightly bound molecules forming the soft corona of a gel-like structure. Because of relatively low stability, soft corona composition and structure are difficult to characterize by available experimental techniques, which require nanoparticle suspension centrifugation or filtration steps. Additionally, the presence of soft coronas, which promote bridging interactions among nanoparticles, may lead to the destabilization of particle suspensions.

It should be underlined, however, that an interesting aspect of protein coronas at nanoparticles was not considered in previous works, namely that such structures can mimic the behavior of coronaviruses such as the SARS-CoV, MERS and recently SARS-CoV-2 [31]. The intact virion structure and physical dimensions of the latter virus were established in [32,33,34] by a variety of experimental techniques comprising cryo-TEM imaging [33,34]. The virus single-stranded RNA genome is encapsidated by the 5 nm thick membrane stabilized by the M and E proteins [31]. However, the most important for the virus proliferation is the spike (S) protein flexibly incorporated into the membrane [33,34,35,36,37], which stabilizes the virus capsid and is responsible for its attachment to the human angiotensin-converting enzyme 2 (ACE2) cell receptor [35,36]. It is also established that the SARS-CoV-2 virions are spherically shaped with the core part diameter equal to 91 nm and the external diameter (with the S protein envelop) of ca 120 nm [34,37].

Coronavirus attachment to abiotic surfaces (fomites) is a crucial issue controlling their inactivation and removal by filtration (various kinds of masks), which can decrease the proliferation of epidemics. However, because of limited availability of intact virus (virions) and restrictive procedures, no systematic experimental works have been devoted to a thorough physicochemical analysis of this process.

It is postulated in this work above that reliable information about the basic mechanisms of virus attachment to abiotic surfaces, particularly about the deposition rate under various conditions, can be acquired using a polymer nanoparticle with a protein corona analogue, which can mimic physicochemical parameters of the virion, especially its size and zeta potential.

Therefore, in this work, attention is focused on the polystyrene nanoparticle/human serum albumin (HSA) conjugate. There are considerable advantages of such a system given that the HSA molecule physicochemical characteristics are known [30]. It is a globular, single α-chain protein consisting of 585 amino acids with molar mass calculated from this amino acid composition of 66,439 g L^−1^ [38,39]. The crystalline structure consists of 69% α-helix and 17 disulfide bonds [30,38]. Although its shape is rather irregular, it can be approximated by an ellipsoid with the dimensions of 9.5 × 5 × 5 nm [40]. In Refs. [30,41], a coarse-grained HSA molecule model was developed allowing to quantitatively predict the maximum coverage of the molecule at various substrates comprising polymer microparticles.

As far as electrokinetic properties are concerned, the isoelectric point of the HSA molecule was determined to be 5.1 [40], which indicates that it exhibits a positive net charge at pH < 5.1 [38,42]. This property was exploited in this work to prepare HSA coronas at negatively charged polymer particles in a self-assembly process driven by electrostatic interactions. The corona formation was monitored under in situ conditions via the electrophoretic mobility measurements, which was converted to the zeta potential. In the next step, the deposition mechanism of the particles on different surfaces was thoroughly investigated, which represents the main goal of this work.

It is expected that through exploiting these experimental data one can develop a robust procedure for preparing stable virus-like particle suspensions, characterized by well-defined physicochemical characteristics, which can be used for modelling the kinetics of virus deposition on abiotic surfaces and for their efficient removal by various filtration procedures.

## 2. Materials and Methods

The experimental data presented hereafter were obtained for the human serum albumin (HSA) in the form of a lyophilized powder 99% (Sigma-Aldrich (Merck) St. Louis, MO, USA) having the fatty acid content of 0.02% [43,44].

All chemical reagents such as sodium chloride and hydrochloric acid were commercial products of Sigma Aldrich and were used without additional purification. Ultrapure water was obtained using the Milli-Q Elix&Simplicity 185 purification system from Millipore (Darmstadt, Germany).

The effective bulk concentration of albumin molecules in the stock solutions was spectrophoretically determined according to the procedure described in Refs. [44,45]. The stock albumin solution was diluted to a desired concentration before each experiment.

Suspensions of negatively charged polystyrene particles bearing sulfate surface groups and positively charged amidine particles were commercial products of Invitrogen (Life Technologies Polska Sp. Z o.o., Warsaw, Poland). The stock suspension of the 4% concentration was purified by a thorough membrane filtration and diluted to a desired concentration before each experiment.

Natural ruby mica (Continental Trade, Warsaw, Poland) was used as a solid substrate for the HSA adsorption studied by atomic force microscopy (AFM) imaging. Thin sheets of mica were freshly cleaved before each experiment and used without any pretreatment.

The diffusion coefficients of HSA molecules and polymer particles were determined by dynamic light scattering (DLS) using the Zetasizer Nano ZS (Malvern, Cambridge, UK). The hydrodynamic diameters were calculated from the Stokes–Einstein equation.

The electrophoretic mobility of protein molecules and the polymer particles were measured by the laser Doppler velocimetry (LDV) technique using the Zetasizer Nano ZS. The zeta potential was calculated using the Henry and Smoluchowski formulae, respectively.

Atomic force microscopy (AFM, Moscow, Russia) measurements were carried out using the NT-MDT OLYMPUS IX71 device with the SMENA scanning head. The measurements were performed in semi-contact mode using silicon probes and polysilicon cantilevers HA-NC ETALON with resonance frequencies of 140 kHz ± 10% or 235 kHz ± 10%.

In the DLS and the LDV measurements, HSA solutions of the concentration of 500 mg L^−1^ were used. In the adsorption experiments investigated by AFM they were diluted to 0.1–0.5 mg L^−1^, and in the corona formation experiments the initial concentration range of the protein was equal to 1−30 mg L^−1^.

The quartz microbalance (QCM) deposition kinetic experiments were carried out according to the standard procedure described in Refs. [46,47,48]. Initially, a stable baseline for the pure electrolyte (NaCl) at fixed ionic strength and pH was obtained. Afterward, the particle suspension was flushed through the cell at a fixed flow rate. After a prescribed time, the pure electrolyte solution of the same pH and ionic strength was flushed in order to study particle desorption.

Gold sensors used in these experiments were a commercial product of Q-Sense, Gothenburg, Sweden. The sensors were cleaned before each experiment in a mixture of 95% sulfuric acid (H_2_SO_4_) and hydrogen peroxide (30%) in volume ratio of 3:2 for 10 min. Afterward, the sensor was rinsed by deionized water at 80 °C for 30 min and dried out in a stream of nitrogen gas. The roughness of sensors was examined by semi-contact mode of atomic force microscopy (AFM) imaging carried out under ambient conditions. It was confirmed that the sensors were smooth, exhibiting the root mean square roughness of 1.0 ± 0.2 nm.

The deposited particle mass per unit area, hereafter referred to as the QCM coverage, was calculated from the Sauerbrey equation [48,49]

(1) $$\Gamma_{QCM}=-C_{Q}\frac{\Delta f}{n_{o}}$$

where Δf is the frequency change, *n_o_* is the overtone number and *C_Q_* is the mass (coverage) sensitivity constant equal to 0.177 mg m^−2^ Hz^−1^ for the 5 MHz AT-cut quartz sensor [49,50,51,52].

The pH in the range of 3 to 5 was adjusted by the addition of HCl, the pH of 7.4 was fixed by the PBS buffer and larger pHs were adjusted by NaOH.

The temperature of experiments was fixed at 298 ± 0.1K.

## 3. Results and Discussion

### 3.1. Physicochemical Characteristics of Albumin and Polymer Particles

Initially, basic physicochemical characteristics of HSA and the polymer particle suspensions were acquired applying the DLS, LDV and AFM measurements.

**Figure 1:**
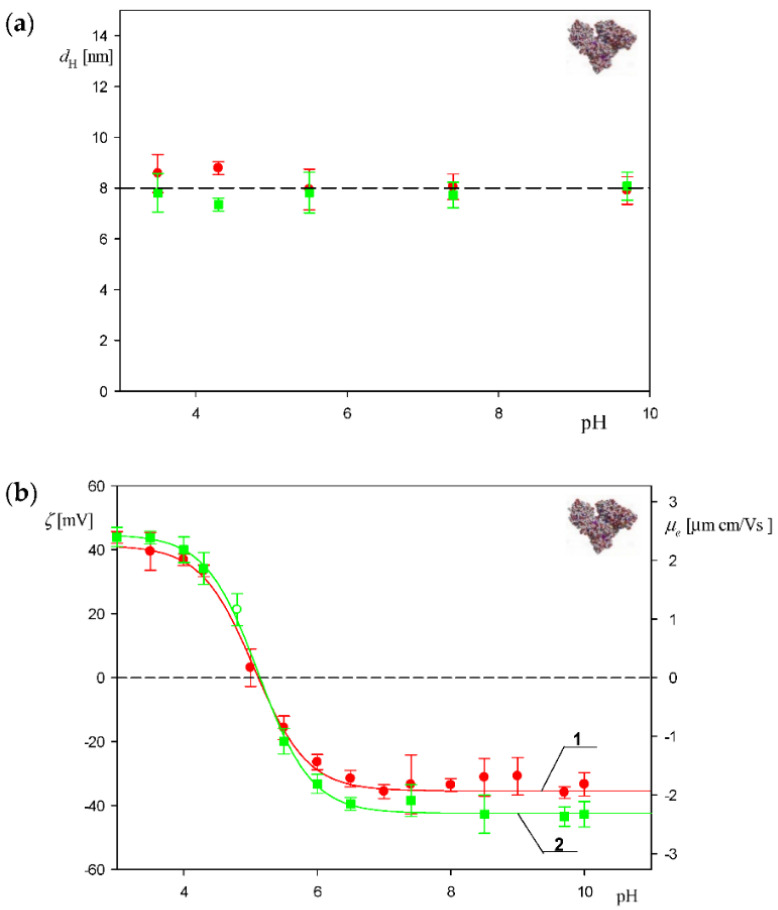
(**a**) Dependence of the hydrodynamic diameter of the HSA molecule on pH derived from the DLS measurements: (■) 1 mM NaCl, (●) 10 mM NaCl. The line shows the average value of the hydrodynamic diameter equal to 8.0. (**b**) Dependence of the zeta potential of the HSA molecules (left-hand axis) and the electrophoretic mobility (right-hand axis) on pH derived from the LDV measurements: 1. (●) 10 mM NaCl, 2. (■) 1 mM NaCl. The lines represent the guide for the eyes.

The average value of the diffusion coefficient of HSA molecules measured by DLS for 300 mg L^−1^ solutions and pH range of 3.5–9 was equal to 6.2 ± 0.3 × 10^−7^ cm^2^ s^−1^. This corresponds to the hydrodynamic diameter calculated from the Stokes–Einstein relationship equal to 8.0 ± 0.4 nm (see Figure 1a). It should be mentioned that the diffusion coefficient remained unchanged within the time period up to 180 min, which considerably exceeds the time in the corona formation measurements.

The electrophoretic mobility of albumin molecules and the particles was measured as a function of pH using the LDV method for 1 and 10 mM NaCl solution concentration. Using these data, the zeta potential was calculated using the Henry equation. The results shown in Figure 1b indicate that the zeta potential of the HSA molecules at pH 3.5 in 10 mM NaCl was equal to 40 ± 2 mV and rapidly decreased to zero and pH 5. At pH 7.4 the zeta potential attained a negative value of −40 ± 2 mV.

**Figure 2:**
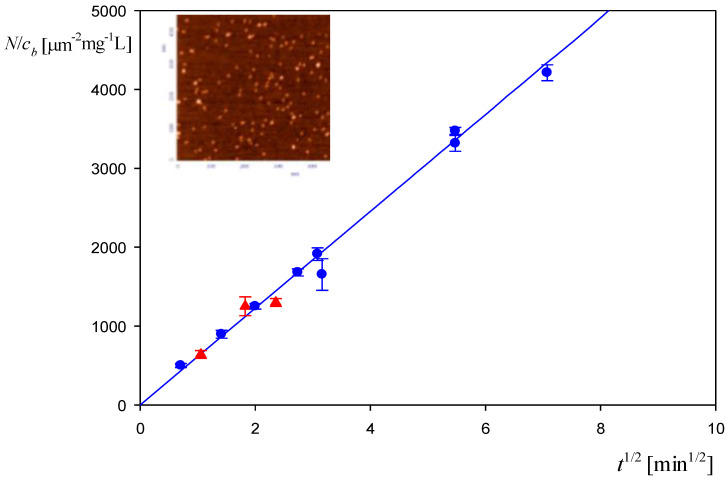
Dependence of the normalized surface concentration of HSA, *N/c_b_* [μm^−2^ mg^−1^ L], on the square root of the adsorption time *t*^1/2^ [min^1/2^]. The points denote experimental results obtained by a direct atomic force microscopy (AFM) enumeration of adsorbed HSA molecules on mica: pH 3.5, 10 mM NaCl, (▲) *c_b_* = 0.1 mg L^−1^, (●) *c_b_* = 0. 5 mg L^−1^. The solid line shows the theoretical results calculated from Equation (2). The inset shows the AFM image of the HSA layer on mica.

Additional information about the albumin solution stability and its diffusion coefficient was acquired via adsorption kinetic measurements carried out using atomic force microscopy (AFM) with single-molecule enumeration according to the procedure described in Ref. [30]. The adsorption runs were carried out under diffusion transport for the bulk protein concentration equal to 0.1 and 0.5 mg L^−1^ at pH 3.5 and 10 mM NaCl concentration. The progress of protein adsorption was quantified in terms of the surface concentration of molecules denoted by *N* and expressed as the number of molecules per square micrometer. As shown in Figure 2, the experimental data are well described by the following dependence:

(2) $$N/c_{b}=2\times 10^{-14}\frac{N_{Av}}{M_{w}}{\left(\frac{D}{\pi }\right)}^{\frac{1}{2}}{\left(60\;t\right)}^{\frac{1}{2}}$$

where *c_b_* is the mass concentration of albumin in the bulk expressed in mg L^−1^, $N_{Av}$ is the Avogadro constant, *M_w_* is the molar mass of the protein expressed in g, *D* is the diffusion coefficient (cm^2^ s^−1^) and *t* is the adsorption time (minutes).

This quantitative agreement between experimental data and the theoretical formula confirms that the albumin solutions were stable, and that the diffusion coefficient was precisely determined by DLS. These findings enable an adequate interpretation of the corona formation experiments discussed in the next section.

**Figure 3:**
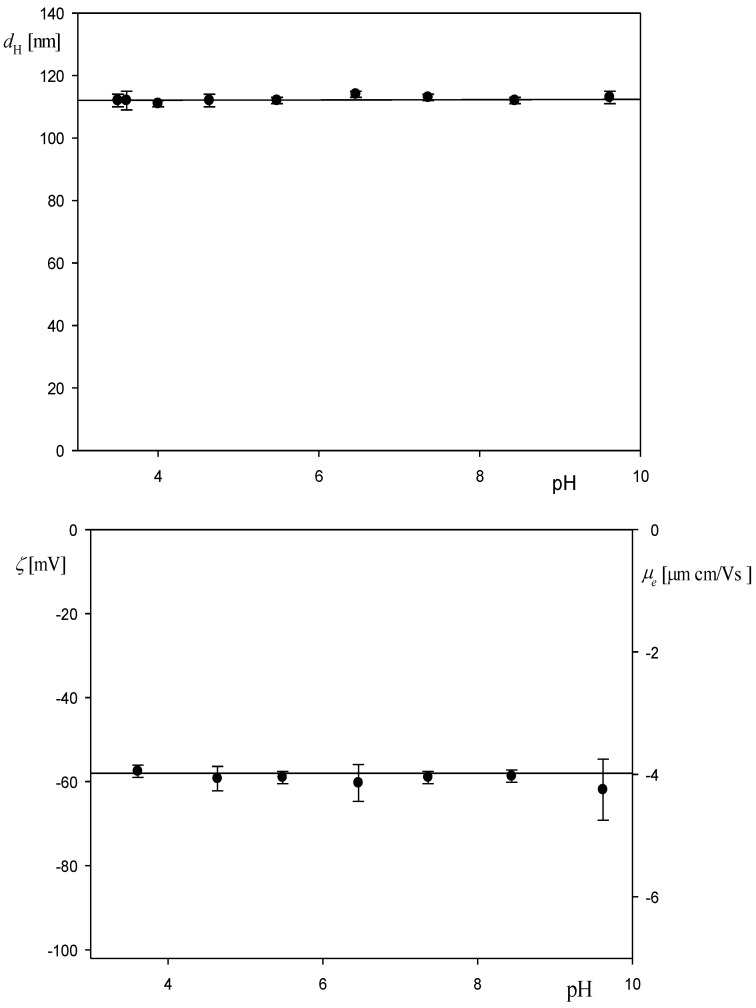
(**a**) Dependence of the hydrodynamic diameter of the polystyrene particles on pH: (●) 10 mM NaCl. The dashed line shows the average value of the hydrodynamic diameter equal to 115 ± 5 nm. (**b**) Dependence of the zeta potential of the particles on pH (left-hand axis) and the electrophoretic mobility (right-hand axis): (●) 10 mM NaCl. The solid line shows the average value of the zeta potential equal to −55 ± 5 mV.

Analogous physicochemical characteristics were carried out for the polymer particles. The results shown in Figure 3a,b indicate that the hydrodynamic diameter of the polystyrene particles at pH 3.5 and 10 mM NaCl was equal to 115 ± 5 nm and remained practically constant for pH up to 9. An analogous behavior was experimentally observed for the zeta potential (calculated using the Smoluchowski formula exploiting the electrophoretic mobility data derived from LDV measurements), which was equal to −55 mV for the pH range 3.5 to 9.

On the other hand, the hydrodynamic diameter and the zeta potential of the amidine particles were equal to 110 ± 5 nm and 60 mV for 10 mM NaCl and the pH range 3.5–9.

**Figure 4:**
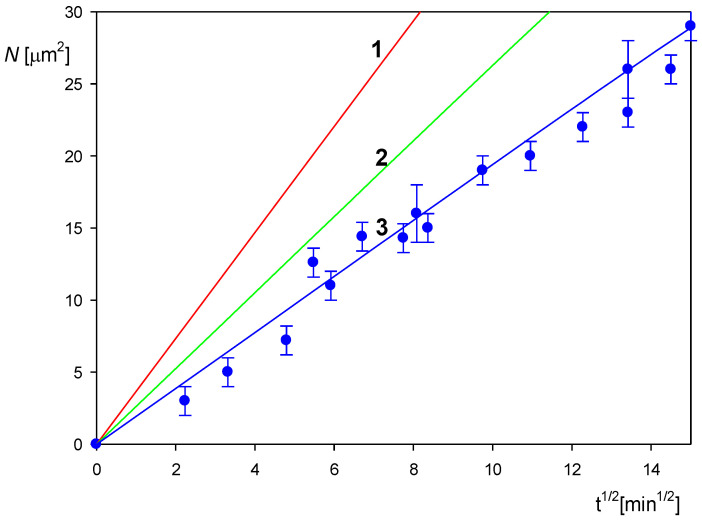
Kinetics of the polystyrene particle deposition on PLL functionalized mica under diffusion conditions shown as the dependence of surface concentration *N* [μm^−2^] on the square root of the deposition time *t*^1/2^ [min^1/2^]. The points denote experimental results obtained by a direct AFM enumeration for *c_p_* = 100 mg L^−1^, pH 3.5 and 10 mM NaCl. The solid lines show the theoretical results derived from Equation (3) for the particle diameter equal to the following: 1. 100 nm, 2. 110 nm, 3. 120 nm.

As previously stated, additional information about the particle physicochemical characteristics was derived from the deposition kinetic measurements performed under diffusion conditions applying the direct AFM molecule enumeration method. The results are shown in Figure 4 as the dependence of the particle surface concentration on the square root of the deposition time. The experimental data are well described for the time up to 200 min by Equation (2) transformed to the following form:

(3) $$N=1.2\times 10^{-13}\frac{1}{\pi \rho_{p}d_{p}^{3}}{\left(\frac{D_{p}}{\pi }\right)}^{\frac{1}{2}}{\left(60\;t\right)}^{\frac{1}{2}}c_{p}=1.2\times 10^{-13}\frac{1}{\pi^{2}\rho_{p}d_{p}^{7/2}}{\left(\frac{kT}{3\eta }\right)}^{\frac{1}{2}}{\left(60\;t\right)}^{\frac{1}{2}}c_{p}$$

where $\rho_{p}$ is the particle density, *dp* is the particle size (hydrodynamic diameter), $D_{p}=\frac{kT}{3\pi \eta d_{p}^{}}$ is the particle diffusion coefficient (*k* is the Boltzmann constant, *T* is the absolute temperature and *η* is the dynamic viscosity of the solution) and *c_p_* is the particle mass concentration in the bulk.

Equation (3) indicates that the surface concentration rapidly decreases with the particle size, proportionally to $d_{p}^{-7/2}$, which suggests that the kinetic measurements can be used for a precise determination of the particle size. This is illustrated in Figure 4, where the theoretical results calculated from Equation (3) for various particle diameters are compared with the experimental results derived from AFM. The best fit is obtained for the particle size of 120 nm, which agrees within the error bounds with the hydrodynamic diameter derived from AFM.

### 3.2. Formation of Albumin Corona on Polymer Particles

Albumin adsorption at polymer particles leading to corona formation was carried out according to the procedure described in Refs. [28,30]. Initially, the protein solution of the concentration varied between 1 and 30 mg L^−1^ and was mixed with the suspension of microparticles of the fixed bulk concentration of 500 mg L^−1^ and incubated over a few minutes at room temperature. Afterward, the electrophoretic mobility of the protein-covered particles was measured. In this way, the dependence of the electrophoretic mobility of the particles on the amount of added protein was directly obtained. This procedure was reproducible, enabling to derive in a reliable way dependencies of the electrophoretic mobility and the zeta potential of particles on the initial protein concentration in the suspension. Another advantage of the procedure consists in the fact that the characteristic time of corona formation at polymer microparticles was significantly shorter compared to the adsorption at planar substrates and can be calculated from the formula [53]

(4) $$t_{c}=\frac{{\left[{\left(\phi_{v}/\phi_{mx}\right)}^{-1/3}-1\right]}^{2}d_{p}^{2}}{4\bar{D}}$$

where $\phi_{v}=c_{p}/\rho_{p}$ is the volume fraction of the polymer particle suspension, $\phi_{mx}$ is the maximum volume fraction equal to 0.62 for a quasi-random packing of spheres and $\overset{-}{D_{}}$ is the mutual diffusion coefficient of albumin molecules relative to the particle.

Using the parameters pertinent to our measurements, i.e., *c_p_* = 500 mg L^−1^, $\rho_{p}$ = 1.05 g cm^−3^, *d_p_* = 115 nm and $\overset{-}{D_{}}$ = 6.2 × 10^−7^ cm^2^ s^−1^, one obtains from Equation (4) that *t_c_* = 0.004 s, which is considerably shorter than the experimental incubation time.

**Figure 5:**
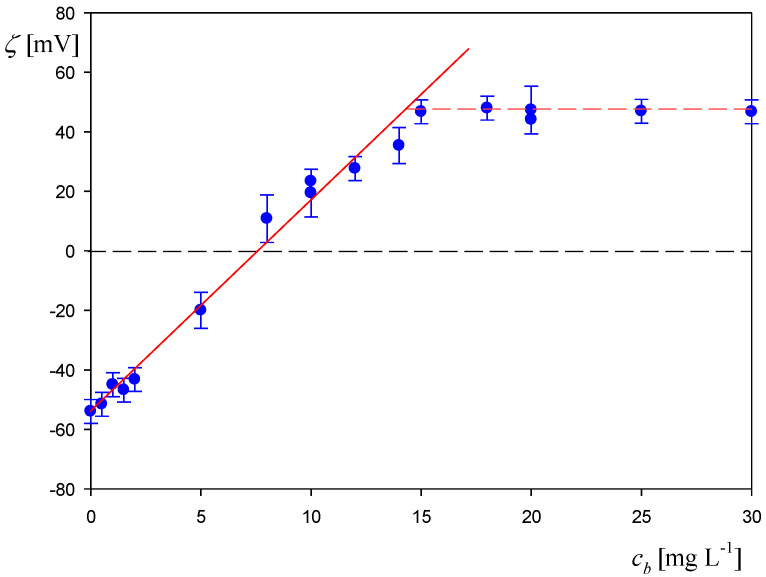
Dependence of the zeta potential of the polystyrene particles on the HSA concentration in the suspension before mixing: pH 3.5, 10 mM NaCl, particle concentration 500 mgL^−1^, (●) experimental results derived from the LDV measurements. The solid red line shows the linear fit of experimental data, and the dashed horizontal line shows the zeta potential of the protein in the bulk.

In Figure 5 the dependence of the zeta potential of the polystyrene particles on the initial concentration of HSA in the suspension acquired via the LDV measurements for pH 3.5 and 10 mM NaCl is presented. The zeta potential rapidly increases with the albumin concentration and becomes positive for *cb* larger than ca. 10 mg L^−1^. For larger concentration above 15 mg L^−1^ a plateau value of the zeta potential is attained, matching the bulk zeta potential of HSA molecules equal to 40 mV. The experimental data were adequately fitted by the straight line described by the equation

(5) $$\zeta (mV)=-58+6.5c_{b}$$

Using Equation (6) the maximum coverage of HSA in the corona formed at the polymer particles can be estimated by observing that the intersection point of this line with the horizontal one representing the bulk zeta potential of albumin occurs at $c_{b}$ = 15 mg L^−1^. Using this concentration, one can calculate the mass coverage of the protein corona denoted as $\Gamma_{c}$ from the following dependence [29,30]

(6) $$\Gamma_{c}=\left(\frac{\rho_{p}d_{p}}{6}\right)\frac{c_{b}}{c_{p}}$$

which was equal to 0.70 mg m^−2^.

It should be mentioned, however, that the precision of the maximum coverage determination via the LDV method does not exceed 0.1 mg m^−2^. A more precise estimation can be attained applying the AFM-aided method where the residual albumin coverage in the suspension after forming the corona was quantitatively determined via adsorption kinetic measurements according to the method described in Ref. [30]. It is determined in this way that the maximum corona coverage was equal to 0.80 ± 0.05 mg m^−2^, which corresponds to the initial concentration of the albumin in the suspension of 17 mg L^−1^. Additionally, applying the AFM method, the average size of the particles with the HSA corona deposited on mica was determined. It was equal to 130 ± 5 nm, compared to the above value of 115 ± 10 nm pertinent to bare particles derived from DLS measurements. The difference of these two values indicates that the thickness of the HSA corona on the polymer particles was equal to 7.5 nm, which agrees within experimental error bounds with the size (hydrodynamic diameter) of the albumin molecule.

**Figure 6:**
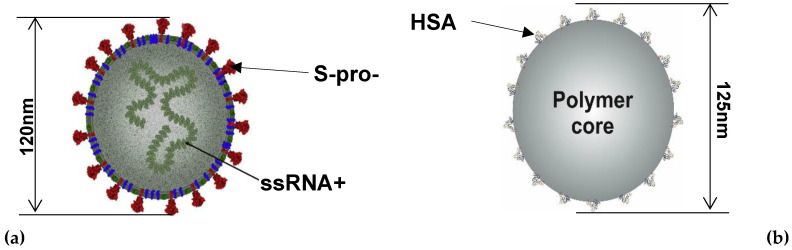
(**a**) A schematic view of the SARS-CoV-2 virion [37] and (**b**) the polymer particle with the HSA corona.

The stability of the polymer particles with HSA coronas in electrolyte solutions was determined measuring by DLS their diffusion coefficient at various pHs. Afterward, these data were converted to the hydrodynamic diameter using the Stokes–Einstein formula. It was observed that the particles with the corona coverage of 0.80 mg m^−2^ were stable for pH below 5 over the time exceeding 24 h, and their hydrodynamic diameter was equal to 125 ± 10 nm, which agrees with the particle size derived from AFM. Interestingly, this is close to the external diameter of the SARS-CoV-2 intact virion [32,33,34,36,37] that is schematically shown in Figure 6.

**Figure 7:**
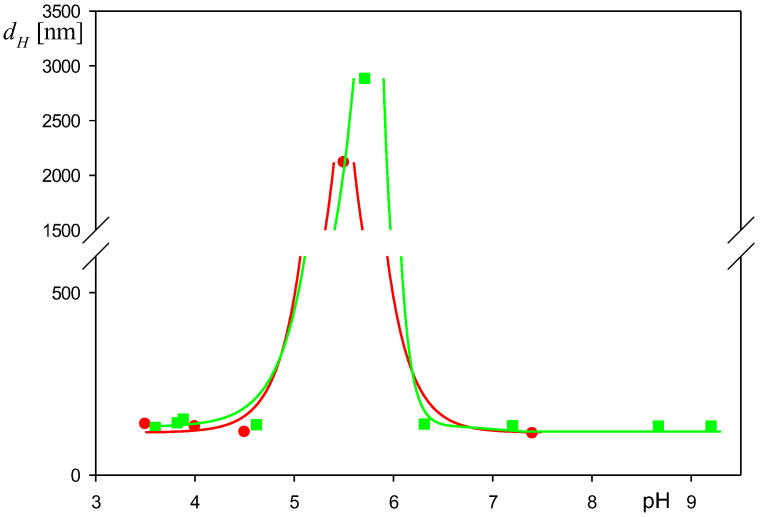
Dependence of the hydrodynamic diameter of the particles with the HSA corona on pH: corona coverage 0.80 mg m^−2^, (●) 10 mM NaCl, (■) 1 mM NaCl. The lines represent guides to the eyes.

However, at pH within the range of 5 to 6.5, the hydrodynamic diameter of the particles significantly increased (see Figure 7), which suggests a low stability of their suspensions.

It was also confirmed in another series of experiments that the particles with the corona coverage below 0.5 mg m^−2^ showed lower stability for all pHs. Therefore, in the experiments described below the corona coverage was kept at the constant level of 0.80 mg m^−2^.

**Figure 8:**
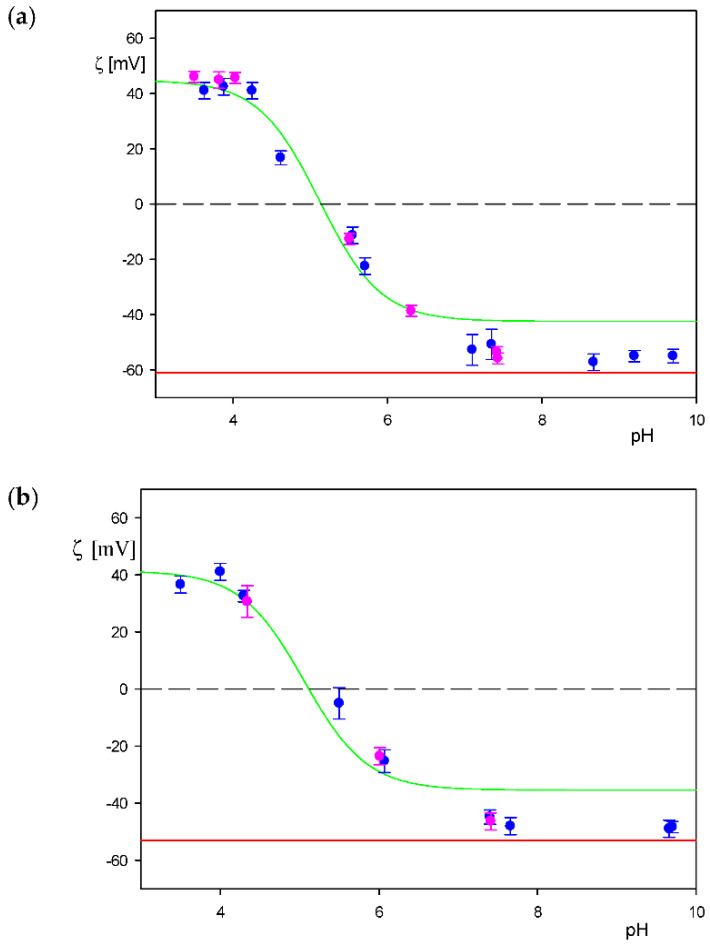
Dependencies of the zeta potential on pH derived from the LDV measurements: (**a**) 1 mM NaCl, (**b**) 10 mM NaCl. The green lines show the bulk zeta potential of HSA molecules, the red lines show the bulk zeta potential of the bare polymer particles and (●,●) represents experimental data for the particles with the HSA corona (the coverage of 0.80 mg m^−2^).

Additionally, to properly interpret the deposition kinetic measurement, the dependencies of the particle zeta potential on pH were determined for the NaCl concentrations of 1 and 10 mM. The results shown in Figure 8 indicate that for both NaCl concentrations the particle zeta potential coincided with the albumin molecule zeta potential in the bulk (marked by the green line) for pH below 7. Therefore, the isoelectric point of the particles acquired from these measurements, equal to 5.0 ± 0.2, is similar to the HSA molecule isoelectric point of 5.1 [30,40]. It is interesting to mention that these results, showing that the zeta potential of the particles becomes negligible around pH of 5, explain their low stability at this pH (see Figure 7).

### 3.3. Deposition of the Particles with the HSA Corona

**Figure 9:**
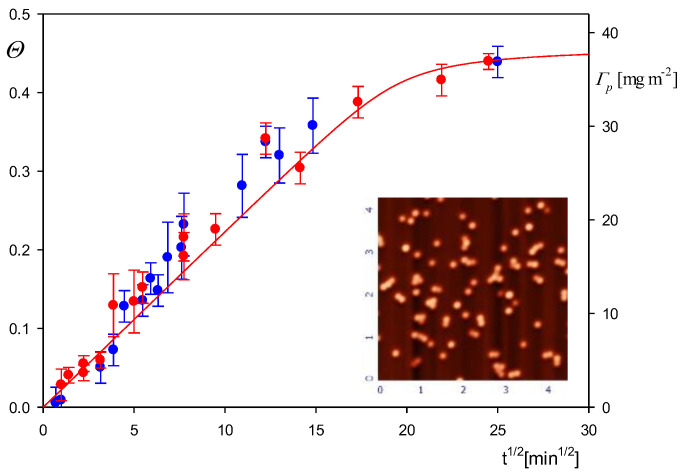
Kinetics of the polymer particle with HSA corona deposition on bare mica under diffusion conditions shown as the dependence of the absolute coverage Θ (left-hand axis) or the mass coverage $\Gamma_{p}$ (right-hand axis) on the square root of the deposition time *t*^1/2^ [min^1/2^]: the corona coverage 0.80 mg m^−2^, pH 4, 10 mM NaCl, particle bulk concentration 100 mg L^−1^. The red points show experimental results obtained by AFM imaging of particles. The inset shows the AFM image of the particle with the HSA corona layer of mica. The blue points show the reference results obtained for the positively charged polymer particles. The solid red line shows the theoretical results derived from the random sequential adsorption (RSA) model.

After establishing the physicochemical properties of the particles with HSA corona thorough measurements were carried out with the aim of determining their deposition kinetics at different abiotic substrates under the diffusion- and convection-controlled transport. In Figure 9 the results obtained for the mica substrate under diffusion conditions are presented as the dependence of the absolute coverage *Θ* = *N S_g_* (where *S_g_* is the cross-section area of the particle) on the square root of the deposition time. For comparison, the particle mass coverage $\Gamma_{p}$ = *N m*_1_ = *Θ m*_1_/*S_g_* (where *m*_1_ is the mass of a single particle) is also presented in the right-hand axis of Figure 9. It should be remembered that the surface concentration *N* was determined by a direct AFM enumeration of the deposited particles. This procedure was reliable because no information about the particle size, shape and distribution over the substrate surface is needed. One can observe in Figure 9 that for *t*^1/2^ below 15 (i.e., for the adsorption time equal to 225 min) the particle coverage linearly increases with the square root of the deposition time, which confirms that the surface blocking effects stemming from interactions among deposited particles play a negligible role. At a longer deposition time, the plateau value of the particle coverage equal to 0.43 (this corresponds to $\Gamma_{p}$ = 38 ± 2 mg m^−2^) was asymptotically attained. Interestingly, almost identical results were obtained for the positively charged amidine particles depicted as blue points in Figure 9.

These results were theoretically interpreted in terms of the general random sequential adsorption (RSA) model described in Ref. [52], where the coupling of the bulk transport of particles governed by diffusion with the surface layer transport governed by the blocking effects was considered in an exact way. The resulting boundary value problem was numerically solved by the finite difference implicit Crank–Nicholson method.

As shown in Figure 9, the theoretical results stemming from the RSA model adequately reflect the experimental data for the entire range of the deposition time, which indicates that the particle deposition rate attained the maximum value feasible for barrier-less transport conditions [52]. Hence, these results can be used as reference data for the interpretation of experiments performed by quartz microbalance QCM under flow conditions where the particle mass can only indirectly be determined.

Additionally, the particle layer topography was characterized in terms of the root mean square (*rms*) parameter determined as a function of the coverage using the AFM software. The experimental *rms* determined in this way monotonically increased with the coverage from 31 ± 2 to 54 ± 2 nm for *Θ* = 0.1 and 0.43, respectively. These experimental data were compared with theoretical values calculated from the formula [53]

(7) $$rms=0.841{\left[\Theta \left(1-0.98\Theta \right)\right]}^{1/2}d_{p}^{}$$

It is predicted from this equation that the particle layer *rms* should be 32 and 53 nm for *Θ* = 0.1 and 0.45, respectively, assuming the particle diameter of 125 nm, which agrees within experimental error bounds with the experimental data.

**Figure 10:**
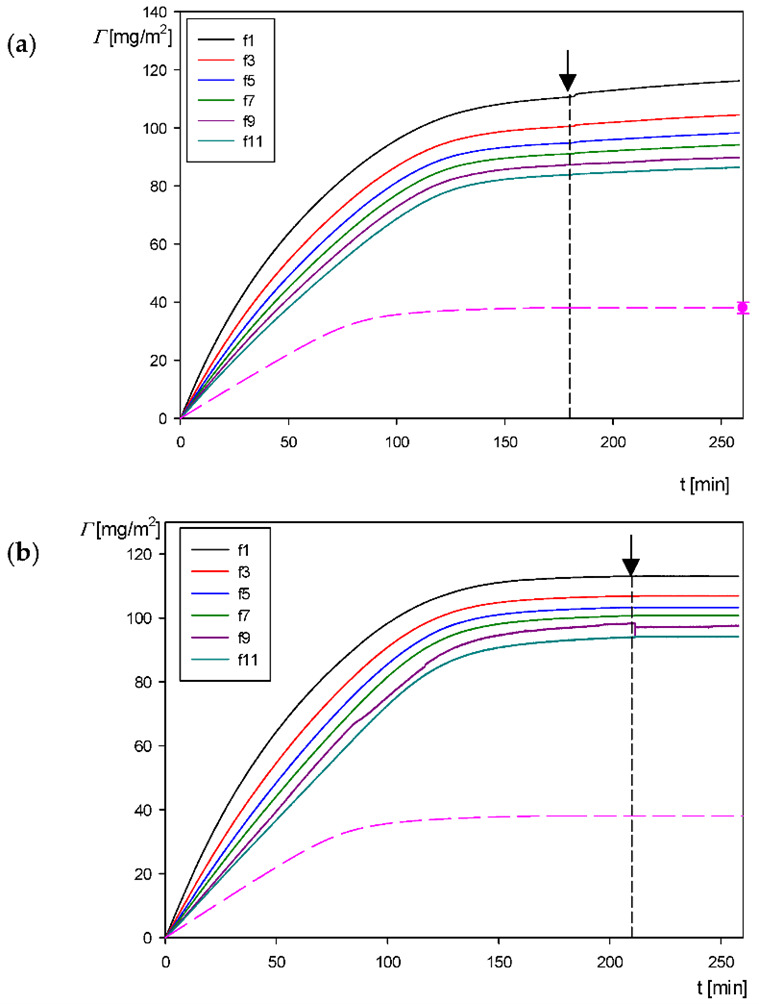
(**a**) Kinetics of the polymer particle deposition on the gold sensor derived from quartz microbalance (QCM) measurements: the corona coverage 0.80 mg m^−2^, pH 4, 10 mM NaCl, *c_b_* = 50 mg L^−1^, flow rate 2.5 × 10^−3^ mL s^−1^. The solid lines show the experimental data derived from the Sauerbrey model for various overtones, the dashed lines show the results predicted using the RSA model and the point shows the dry particle coverage derived from AFM. (**b**) Reference kinetic results for the positively charged amidine particle deposition on the gold sensor derived from QCM measurements: pH 3.5, 10 mM NaCl, *c_b_* = 50 mg L^−1^, flow rate 2.5 × 10^−3^ mL s^−1^. The solid lines show the experimental data derived from the Sauerbrey model for various overtones and the dashed lines show the results predicted using the RSA model.

On the other hand, information about the particle deposition under flow conditions was derived from QCM performed according to the above-described procedure. Primarily, in these experiments the dependence of the frequency shift on the time was recorded for various overtones. Using Equation (1), these primary signals were converted to the dependence of the QCM (wet) coverage *Γ_Q_* on the deposition time. A typical kinetic run acquired for pH 4, 10 mM NaCl and the bulk particle concentration of 50 mg L^−1^ is shown in Figure 10a. For comparison, in Figure 10b analogous results for the same set of parameters acquired for the bare amidine particles are shown. As can be seen in Figure 10a, the particle coverage abruptly increases with the deposition time, attaining a plateau value of 110 and 80 mg m^−2^ at *t* = 180 min for the 1st and the 11th overtones, respectively. Similar results were obtained for the amidine particles with the plateau coverage of 110 and 90 mg m^−2^ at *t* = 220 min for the 1st and the 11th overtones, respectively (see Figure 10b). It should be mentioned that the decrease in the QCM coverage with the overtone number is a common effect observed for nanoparticles, proteins [54] and viruses [55]. It can be theoretically interpreted in terms of the decrease in the hydrodynamic shearing force acting on the particle layer relative to the inertia force [54].

It was also confirmed in these experiments that upon switching to the pure electrolyte flow (depicted by the arrows in Figure 10), the change in the particle coverage was negligible for all overtones, which can be interpreted as the indication of the lack of particle desorption.

In order to unequivocally interpret the QCM results, the particle coverage at the gold sensor after the desorption run was independently determined by the above-described counting procedure exploiting the AFM images. The absolute coverage obtained in this way was equal to 0.42 ± 0.02 that corresponds to the mass coverage $\Gamma_{p}$ = 37 ± 2 mg m^−2^, which is close to the dry coverage of mica previously determined by AFM for the diffusion-controlled transport equal to 38 ± 2 mg m^−2^. As can be inferred, the maximum QCM coverage is 2.9 and 2.1 times larger (for the 1st and 11th overtones, respectively) than the true dry coverage at the sensor determined by AFM. This observation indicates that the sensitivity of the in situ QCM kinetic measurements is markedly larger (especially for the smaller overtone numbers) than the sensitivity of the tedious AFM measurements.

**Figure 11:**
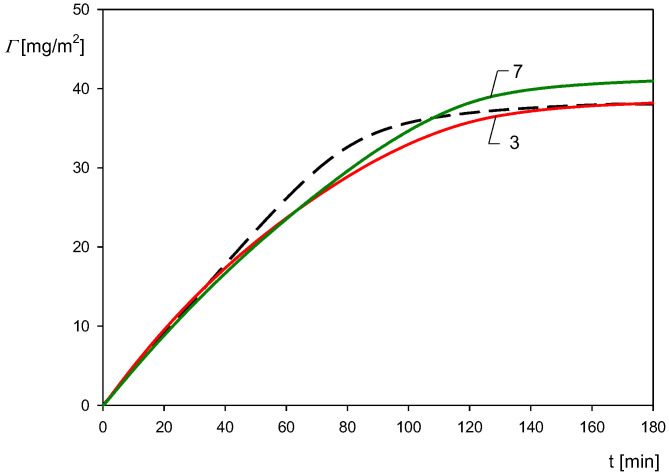
Kinetics of the polymer particle with HSA corona deposition on the gold sensor under flow conditions: the corona coverage 0.80 mg m^−2^, pH 4, 10 mM NaCl, *c_b_* = 50 mg L^−1^, flow rate 2.5 × 10^−3^ mL s^−1^. The solid red line shows the corrected QCM result for the third (red line) and the seventh overtone (green line), and the black dashed shows the theoretical results calculated from the RSA model.

In order to confirm the utility of the QCM method, the kinetic results shown in Figure 10 were compared with theoretical calculations derived from the RSA approach previously applied to interpret nanoparticle deposition kinetics at various substrates [53]. The results obtained from this model are shown as the dashed line in Figure 10. As can be seen, the theoretical coverage *Γ* is considerably smaller than the QCM coverage with the ratio *Γ/Γ_Q_* varying between 0.35 and 0.47 depending on the overtone number (1st and 11th). Considering these quotients, one can transform the QCM apparent coverage to the dry coverage. The results obtained in this way for the third and seventh overtones (with the quotient equal to 0.38 and 0.45 assumed to be independent of the coverage) are shown in Figure 11. As can be seen, the corrected QCM results are well approximated for the entire range of time by the theoretical RSA data, thereby providing useful information about the true deposition kinetic of the particles. This indicates that the QCM results can be applied for comparative studies of particle deposition efficiency under various physicochemical conditions, primarily the suspension pHs.

**Figure 12:**
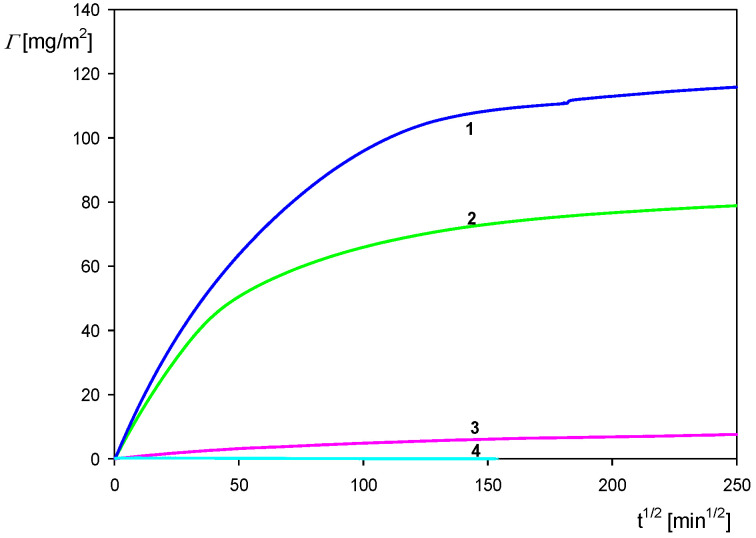
Kinetics of the polymer particle with HSA corona deposition on the gold sensor derived from QCM measurements for various pHs: the corona coverage 0.80 mg m^−2^, 10 mM NaCl, *cb* = 50 mg L^−1^, flow rate 2.5 × 10^−3^ mL s^−1^. The solid lines show the experimental data derived from the Sauerbrey model for the third overtone: 1. pH 4.0, 2. pH = 4.5, 3. pH = 5.5, 4. pH = 7.4.

In Figure 12, QCM results illustrating the influence of pH on the deposition kinetic of the particles with the HSA corona at the gold sensor are shown. One can observe that at pH 4 the kinetics attained the maximum rate; then, at pH 4.5 it significantly decreased, and at still-larger pHs of 5.5–7.4 the kinetic became negligible. Therefore, a direct correlation of the deposition rate with the zeta potential of the particles (see Figure 8) was confirmed in these QCM measurements. This indicates that electrostatic interactions played a decisive role in the deposition of the polymer particles with protein corona.

It is worth mentioning that analogous results were reported in Ref. [55], where systematic QCM studying the deposition kinetics of various bacteriophages (comprising the MS2 virus) on silica and gold sensors modified by self-assembled amine and carboxyl-terminated layers (SAMs) were performed. The intact capsids were spherically shaped with outer diameters of 29 nm. Electrophoretic mobility measurements showed that the capsids exhibited a positive charge for pH below 5 and negative otherwise, analogous to our case for the polystyrene particles with the HSA corona. It was confirmed in Ref. [55] that the deposition kinetics of the MS2 virus at the negatively charged carboxyl-terminated self-assembled monolayer decreased with pH and vanished at pH 6.

## 4. Conclusions

The formation of albumin (HSA) corona at polymer particles as quantitatively evaluated applying the in situ LDV electrophoretic mobility measurements. The stability and physicochemical properties of the particles comprising their zeta potential dependence on pH were also determined. It was confirmed that the particles had a diameter of 125 + 10 nm, which corresponds to the external diameter of the SARS-CoV-2 virion with the spike protein.

The deposition kinetics of the particles was determined by atomic force microscopy under diffusion and by quartz microbalance under laminar flow conditions. It was shown that the particle deposition rate at the gold sensor abruptly vanished at pH above four, which correlated with the zeta potential of the particles. This effect proved that electrostatic interactions played a decisive role in the deposition of the polymer particles with protein corona.

The experimental results acquired in this work showed that it is feasible to prepare stable polymer particles with protein corona (pseudo-virus) suspensions characterized by well-defined physicochemical characteristics. Such particles can be used as reference systems for predicting kinetics of intact virus deposition on abiotic surfaces and for their efficient removal by various filtration procedures.

## Data Availability

The data are available on request. Tested samples are available from the corresponding author (M.N.R.) on request.
